# Plasma Biomarkers, Brain Volume, and Cognitive Performance in Service Members and Veterans With mTBI: A LIMBIC-CENC Study

**DOI:** 10.1001/jamanetworkopen.2025.59596

**Published:** 2026-02-25

**Authors:** Heather E. Dark, Kimbra Kenney, Nicola L. de Souza, Carrie Esopenko, Katie A. Edwards, Emily L. Dennis, Mary R. Newsome, Elisabeth A. Wilde, Jessica M. Gill

**Affiliations:** 1 Johns Hopkins University School of Nursing, Baltimore, Maryland; 2 Department of Neurology, Uniformed Services University of the Health Sciences, Bethesda, Maryland; 3 Department of Neurology, School of Medicine, University of Utah, Salt Lake City

## Abstract

**Importance:**

Repetitive mild traumatic brain injury (mTBI) may result in neurobiological changes that contribute to persistent symptoms of cognitive dysfunction. Elevations in plasma biomarkers (glial fibrillary acidic protein [GFAP], neurofilament light chain [NfL], total-tau [t-tau], and ubiquitin C-terminal hydrolase-L1 [UCH-L1]) have been reported acutely after mTBI, but few studies have assessed these biomarkers during the chronic phase of mTBI.

**Objective:**

To examine whether the associations among plasma biomarkers, brain volume, and cognition varied by mTBI among US active-duty service members and veterans.

**Design, Setting, and Participants:**

This cross-sectional study used data from the Long-Term Impact of Military-Relevant Brain Injury Consortium–Chronic Effects of Neurotrauma Consortium (LIMBIC-CENC), a prospective, multicenter study of US active-duty service members and veterans (SMVs). Data collection began in January 2015, and only enrollment data (collected 2015 to September 2023) were analyzed. Recruitment occurred across several different VA medical centers and military sites across the United States. Participants were aged 18 years or older; had a history of deployment in Operation Enduring Freedom, Operation Iraqi Freedom, or Operation New Dawn; and had combat exposure during deployment. Exclusion criteria were major neurologic or psychiatric disorder, history of moderate to severe TBI, coma lasting more than 30 minutes, posttraumatic amnesia lasting more than 24 hours, and intracranial lesion on computed tomography scan.

**Exposure:**

History of blast- or combat-related mTBI.

**Main Outcomes and Measures:**

Plasma biomarkers were analyzed using Quanterix SIMOA assays. History of mTBI was ascertained using structured clinical interview. Regional brain volume was derived from T1-weighted magnetic resonance imaging scans. Cognitive performance was assessed using neuropsychological measures. Correlations were computed for associations between mTBI, brain volume, and cognition, and separate covariate-adjusted linear regression models we used to examine the association between each plasma biomarker and brain volume as well as cognition. Additional linear regression models were used to examine whether the number of combat-related and blast-related mTBIs moderated the association between each plasma biomarker, brain volume, and cognition.

**Results:**

The cohort included 1160 participants (mean [SD] age, 41.9 [10.2] years; 1025 [88.4%] male; mean [SD] years since last mTBI, 12.1 [9.5]). Less than half of the sample was exposed to at least 1 blast-related (475 [40.9%]) or combat-related mTBI (490 [42.2%]). The association between enrollment biomarker concentration and brain volume and cognitive performance differed by the number of mTBIs. Among those with 2 mTBIs, higher UCH-L1 concentrations were associated with smaller anterior cingulate volume (blast-related mTBI: b = −0.08; 95% CI, −0.16 to −0.01; *P* = .04; combat-related mTBI: b = −0.09; 95% CI, −0.17 to −0.01; *P* = .03). Among those with 2 blast-related mTBIs, higher t-tau was associated with poorer performance on immediate and delayed visual memory (eg, performance on the Brief Visuospatial Memory Test–Revised, immediate recall: b = −0.11; 95% CI, −0.19 to −0.04; *P* = .003; delayed recall: b = −0.08; 95% CI, −0.16 to −0.01; *P* = .03), and higher GFAP and NfL concentration were associated with worse executive functioning (performance on Trail Making Testing B: GFAP concentration: b = 0.20; 95% CI, 0.03 to 0.37; *P* = .02; NfL concentration, b = 0.14; 95% CI, 0.01 to 0.26; *P* = .04), while no biomarker-cognition associations were observed for those with no mTBIs. However, most findings did not pass multiple comparison correction and should be interpreted with caution.

**Conclusions and Relevance:**

In this cross-sectional study of 1160 SMVs, plasma biomarkers of neuronal injury and astrogliosis were associated with brain volume and cognitive performance based on mTBI.

## Key Points

**Question:**

Does the association between plasma biomarkers and brain volume and that between plasma biomarkers and cognition vary by number of combat- and blast-related mTBIs?

**Findings:**

This cross-sectional study of 1160 service members and veterans found that among those with 0 and 2 mTBIs, higher UCH-L1 concentrations were associated with smaller anterior cingulate cortex volume. Further, among those with 2 mTBIs, higher concentrations of biomarkers of neuronal injury were associated with visual memory and executive functioning, while no associations were observed for those with 0 mTBIs.

**Meaning:**

In this study, blood-based biomarkers of neuronal injury were associated with brain structure and cognition, but few results passed multiple comparison correction and should be interpreted with caution.

## Teaser

This cross-sectional study evaluates whether plasma biomarkers of neuronal injury are associated with brain volume and cognition among US active-duty service members and veterans with and without mild traumatic brain injury (mTBI).

## Introduction

Repetitive mild traumatic brain injury (mTBI) is often associated with changes in brain volume,^1^ poorer cognitive performance,^2,3,4^ and subsequent cognitive impairment.^5^ Central structural and physiological changes, including axonal injury, astrocytic reactivity, and blood brain barrier (BBB) dysfunction may underlie the relationship between repetitive mTBI and cognitive and functional outcomes.^6^ Nonspecific plasma biomarkers of neuronal injury (neurofilament light chain [NfL], total tau [t-tau], ubiquitin C-terminal hydrolase-L1 [UCH-L1]) and astrogliosis (glial fibrillary acidic protein [GFAP]) offer a minimally invasive snapshot of neurobiological functioning, increase in response to TBI, and relate to brain volume and cognition.^4,7,8,9,10,11,12^ Few studies have comprehensively examined associations between repetitive mTBI, plasma biomarkers, brain volume, and cognitive performance.

Several studies have examined associations among plasma biomarkers and TBI, especially in the acute (1-7 days) and subacute (7-90 days) phases following injury, with few studies in the remote chronic (>2 years) period. Specifically, GFAP and UCH-L1 concentrations are elevated in moderate and severe TBI within hours after injury^7,8,13^ and differentiate those with TBI from controls.^7,8^ While plasma biomarkers are typically elevated among those with moderate and severe TBI, they show more variability in mTBI.^12^ Time of assessment also affects the association between plasma biomarker concentration and mTBI. For instance, GFAP concentrations differ between mTBI and controls within hours of measurement,^7,9^ but results vary on whether GFAP is elevated or equal between groups within days or months after the injury.^7,8,9^ Similarly, UCH-L1 is elevated and typically differentiates participants with TBI from control participants within hours of injury,^8,9^ but results vary on whether it differs over days to months after injury.^9,14^ Although NfL is often not elevated within hours of injury, it may increase within days to months of injury^7,9^ and decrease years after injury,^12,15^ but remain high compared with controls.^12^ Conversely, others show variable findings of plasma biomarker concentrations in those with TBI vs control participants. When biomarkers are assessed more than 3 months after injury, some studies show no differences in participants with mTBI vs controls.^15,16,17,18^ However, differences in some biomarkers (eg, GFAP) but not others (eg, NfL, t-tau) have been found across all TBI severities.^19^ Studies also demonstrate contradictory findings, where plasma biomarker concentrations are lower in participants with mTBI compared with controls. Specifically, GFAP was found to be elevated in control participants compared with mTBI participants during the remote chronic phase of mTBI.^20,21^ GFAP has also been found to be higher in noninjured control participants than in participants with mTBI when measured during the remote chronic phase.^16^

Repetitive mTBI also varies with brain structure and cognition.^1,2,5,22^ Those with history of mTBI have been shown to have smaller brain volumes,^23^ a thinner cortex,^22^ and faster decline in brain volume^1,24^ as well as unexpected longitudinal increases in brain volume.^25^ Prior studies have also shown that higher UCH-L1,^12^ GFAP,^10,11^ NfL,^26^ and t-tau^12^ relate to smaller brain volumes. However, few studies have linked plasma biomarker concentrations and brain structure in those with mTBI. One study showed that in participants with mTBI, higher t-tau related to both better and poorer white matter organization.^17^ Another study showed that higher NfL and GFAP related to microstructural organization in gray and white matter, and that elevations in NfL related to future white matter volume loss in all TBI severities.^15^ Finally, in those with moderate to severe TBI, acutely assessed UCH-L1, NfL, GFAP, and t-tau related to gray matter atrophy.^27^

While cognitive changes typically resolve within days of mTBI, those with repetitive mTBIs may show lasting cognitive effects. Participants with repetitive mTBIs have shown poorer cognitive performance compared with control participants.^3,4,28,29^ However, this is not always the case.^30^ Further, studies examining whether plasma biomarkers relate to cognition in those with mTBI have demonstrated mixed results. One study showed that plasma biomarkers do not relate to cognition.^17^ Another study found that while plasma biomarkers did not relate to cognition among participants with mTBI, UCH-L1 was positively associated with immediate and delayed memory in participants with severe TBI.^16^ Further investigation is needed to determine associations between plasma biomarkers, brain volume, and cognition in those with repetitive mTBIs. Thus, in the present study we examined (1) whether plasma biomarkers measured during the remote chronic phase of mTBI are associated with brain volume and cognitive performance and (2) whether this association varies by number of mTBIs.

## Methods

### Participants

Enrollment data from the Long-Term Impact of Military-Relevant Brain Injury Consortium–Chronic Effects of Neurotrauma Consortium (LIMBIC-CENC) multicenter study were included in the present cross-sectional study (eFigure 1 in Supplement 1). LIMBIC-CENC recruitment has been previously described.^31,32^ Briefly, service members and veterans (SMVs) were recruited if they were 18 years or older; had a history of deployment in Operation Enduring Freedom, Operation Iraqi Freedom, or Operation New Dawn; and had combat exposure during deployment (ie, a score of >1 on any item of the Deployment Risk and Resilience Inventory–2).^33^ Exclusion criteria included major neurologic or psychiatric disorder, history of moderate to severe TBI (Glasgow coma scale <13), coma lasting more than 30 minutes, posttraumatic amnesia lasting more than 24 hours, or intracranial lesion on computed tomography scan.^31^ Thus, the sample includes those with uncomplicated mTBI. Data collection began in January 2015. Data for the present analyses were collected from 2015 to September 2023. Further details are described in the eMethods in Supplement 1. This article follows the Strengthening the Reporting of Observational Studies in Epidemiology STROBE reporting guidelines for cross-sectional studies. Site-based institutional review boards approved this study. Participants provided written informed consent prior to participation.

### Plasma Biomarkers

UCH-L1, GFAP, NfL, and t-tau concentrations were derived using Quanterix Single Molecule Array (SIMOA) Neurology 4-plex. Further detail is provided in the eMethods in Supplement 1. Due to skeweness of biomarker concentrations, all plasma biomarkers were log2 transformed prior to analyses. No biomarkers were removed due to being an outlier.

### TBI Assessment

Lifetime TBI history was assessed using a modified version of the Ohio State University TBI Identification Method^34^ that included assessing for all potential concussive events (PCEs). Each PCE was then individually assessed to determine whether it met criteria for an mTBI using a Concussive Diagnostic Interview^35^ resulting in a preliminary algorithm-generated TBI diagnosis, which was then reviewed and compared with medical records, and evaluated by an expert committee to ensure diagnoses aligned with Department of Veterans Affairs/Department of Defense–defined mTBI. Further detail is provided in eMethods in Supplement 1.

### Neuropsychological Assessment

Participants completed a comprehensive neuropsychological assessment during their enrollment visit. Cognitive domains include attention/working memory, processing speed, language, memory, and executive functioning. Briefly, the Brief Visuospatial Memory Test–Revised assessed visual learning and memory; the California Verbal Learning Test Second Edition (CVLT-II) assessed verbal learning and memory; letter fluency, category fluency, and the Trail Making Test (TMT) B assessed language and executive function; TMT A and Symbol Search and Coding subsets from the Wechsler Adult Intelligence Scale Fourth edition (WAIS-IV) assessed processing speed, and the Digit Span subset from the WAIS-IV assessed attention/working memory. Further details on these measures and information on self-reported functioning–cognitive performance profiles can be found in the eMethods in Supplement 1.

### Magnetic Resonance Imaging and Brain Volume

Magnetic resonance imaging (MRI) data were acquired using 3T scanners (eTable 1 in Supplement 1). Volumetric segmentation was completed using FreeSurfer image analysis software version 7.4.1. Brain regions of interest included the bilateral rostral anterior cingulate cortex (rACC), caudal anterior cingulate cortex (cACC), middle temporal gyrus (MTG), insula, amygdala, hippocampus, parahippocampal gyrus (PHG), posterior cingulate cortex (PCC), and inferior parietal lobule (IPL). Further detail may be found in the eMethods in Supplement 1.

### Covariates

Covariates included age (years), self-reported race and ethnicity, sex, education (categorized), time since mTBI, and estimated total intracranial volume (eTIV; volumetric analyses only). Race was self-reported, and groups included American Indian or Alaska Native, Asian, Black or African American, Pacific Islander, White or European American, do not know or not sure, and Other, which included individuals who did not self-identify as one of the previously listed groups or chose not to specify.

### Statistical Analysis

Analyses were completed using SPSS statistical software version 25 (IBM Corp) and R version 4.4.2 (R Project for Statistical Computing). Brain volume and cognition were *z* scored prior to analyses. First, Spearman and Pearson correlations were computed to determine associations between mTBI, brain volume, and cognition. Next, separate covariate-adjusted linear regression models were completed to examine the association between each plasma biomarker and brain volume. Third, separate covariate-adjusted linear regression models were completed to examine whether the number of combat-related and number of blast-related mTBIs moderated the association between each plasma biomarker and brain volume. A false discovery rate (FDR) approach was used to correct for multiple comparisons, with FDR-corrected *P* < .05 as the threshold for statistical significance. Further and follow-up analyses are described in the eMethods in Supplement 1.

## Results

### Participant Characteristics

**Figure 1.**
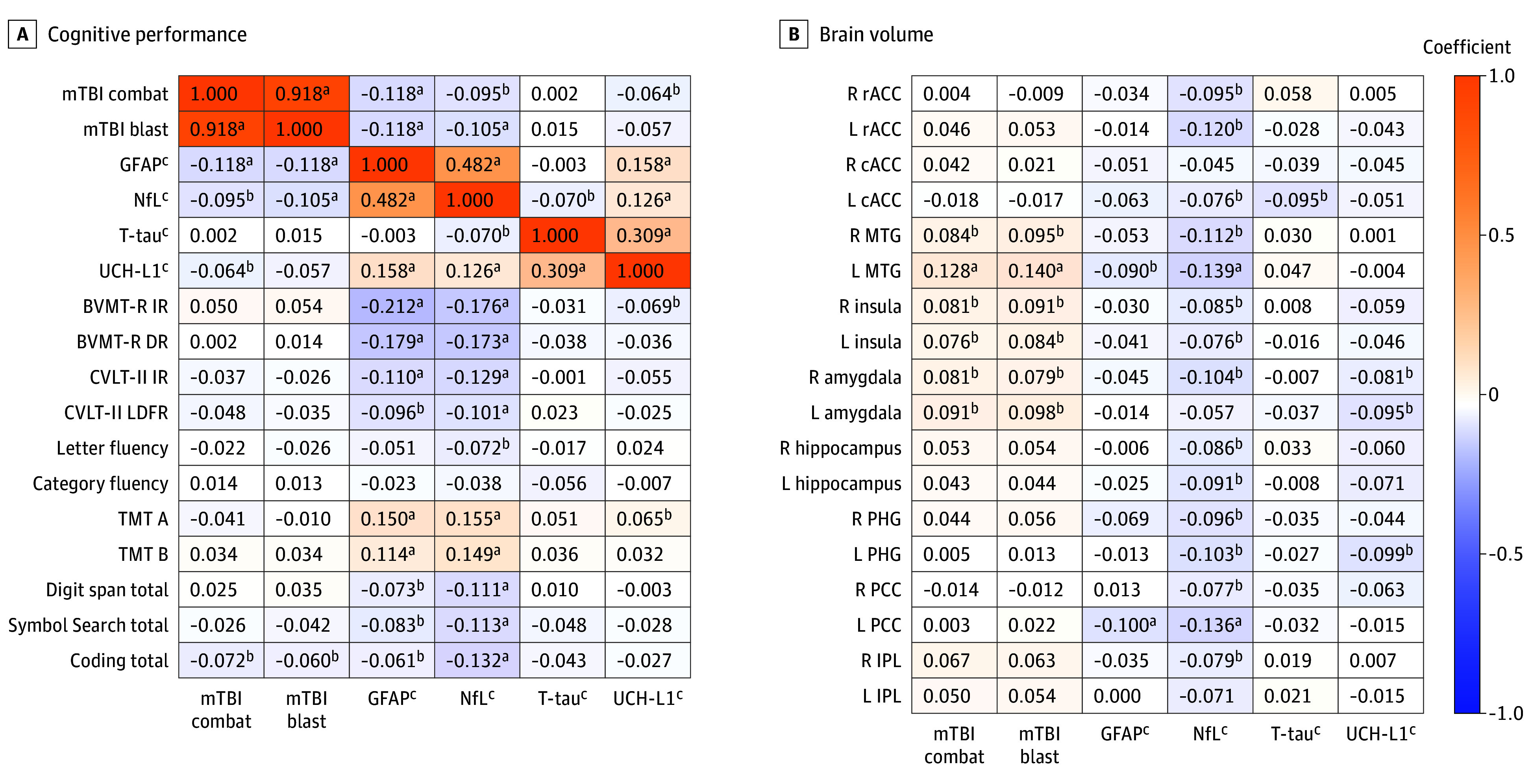
Correlation of Plasma Biomarker Concentrations With mTBI, Cognitive Performance, and Brain Volume A, Spearman ρ and Pearson correlation coefficients between plasma biomarker concentrations, mild traumatic brain injury (mTBI), and cognitive performance (n = 1160 [n = 1159 for analyses including ubiquitin C-terminal hydrolase L1 (UCH-L1)]). B, Spearman ρ and Pearson correlation coefficients between plasma biomarker concentrations, mTBI, and brain volume (n = 698). In both panels, correlations between plasma biomarker concentrations and brain volumes as well as plasma biomarker concentrations and cognitive performance are Pearson correlations, while correlations between mTBI and other variables are Spearman ρ. BVMT-R DR indicates Brief Visuospatial Memory Test–Revised delayed recall; BVMT-R IR, Brief Visuospatial Memory Test–Revised immediate recall; cACC, caudal anterior cingulate cortex; CVLT-II IR, California Verbal Learning Test–II immediate recall; CVLT-II LDFR, California Verbal Learning Test–II long delay free recall; GFAP, glial fibrillary acidic protein; IPL, inferior parietal lobule; L, left; MTG, middle temporal gyrus; NfL, neurofilament light chain; PCC, posterior cingulate cortex; PHG, parahippocampal gyrus; R, right; rACC, rostral anterior cingulate cortex; TMT, Trail Making Test; and t-tau, total tau. ^a^*P* < .001. ^b^*P* < .05. ^c^Data were log2 transformed.

There were 1160 SMVs in the present sample, with a mean (SD) age of 41.9 (10.2) years; 24 (2.1%) Asian participants, 217 (18.7%) Black or African American participants, and 837 (72.2%) White participants; and 1025 (88.4%) male participants. Less than half of the sample was exposed to at least 1 blast-related (475 [40.9%]) or combat-related mTBI (490 [42.2%]). Mean (SD) time since last mTBI was 12.1 (9.5) years (Table 1). However, 9 participants’ (0.8%) time since last mTBI was less than 4 months. Higher GFAP, NfL, and UCH-L1 were associated with fewer combat-related (GFAP: ρ = −0.12; *P* < .001; NfL: ρ = −0.10; *P* = .001; UCH-L1: ρ = −0.06; *P* = .03) and blast-related (GFAP: ρ = −0.12; *P* < .001; NfL: ρ = −0.11; *P* < .001) mTBIs (Figure 1).

**Table 1.** Participant Characteristics

Characteristic	Participants, No. (%) (N = 1160)
Age, mean (SD), y	41.90 (10.22)
Sex	
Male	1025 (88.4)
Female	135 (11.6)
Race	
American Indian or Alaska Native	11 (0.9)
Asian	24 (2.1)
Black or African American	217 (18.7)
Pacific Islander	12 (1.0)
White or European American	837 (72.2)
Other^a^	51 (4.4)
Do not know or not sure	8 (0.7)
Education	
Some college or technical school (1 to 3 y)	451 (38.9)
College graduate (≥4 y)	555 (47.8)
High school graduate/GED	152 (13.1)
Some high school (grades 9 to 11)	2 (0.2)
Blast-related mTBI	
None	685 (59.1)
1	335 (28.9)
2	88 (7.6)
3	33 (2.8)
4	16 (1.4)
5	2 (0.2)
6	0
7	0
8	1 (0.1)
Combat-related mTBI	
None	670 (57.8)
1	342 (29.5)
2	102 (8.8)
3	29 (2.5)
4	15 (1.3)
5	1 (0.1)
6	0
7	1 (0.1)
Time since last mTBI, y^b^	
Mean (SD)	12.14 (9.51)
No mTBI	201 (17.3)
<1	30 (2.6)
≥1 to <3	59 (5.1)
≥3 to <7	202 (17.4)
≥7 to <10	166 (14.3)
≥10	502 (43.3)
T-tau, mean (SD), log2	−0.43 ± 1.38
GFAP, mean (SD), log2	6.18 ± 0.61
NfL, mean (SD), log2	2.79 ± 0.73
UCH-L1, mean (SD), log2^c^	3.31 ± 1.45

Abbreviations: GED, General Education Test; GFAP, glial fibrillary acidic protein; mTBI, mild traumatic brain injury; NfL, neurofilament light chain; t-tau, total tau; UCH-L1, ubiquitin C-terminal hydrolase L1.

^a^ The Other category includes individuals who did not self-identify as one of the listed racial groups or choose not to specify.

^b^ Time since last mTBI categories are reported as they were used for the present study.

^c^ One participant was missing UCH-L1 data.

### Plasma Biomarkers and Brain Volume 

We examined whether enrollment plasma biomarker concentration was associated with enrollment brain volume among 698 SMVs with MRI data. Higher t-tau was associated with smaller brain volumes in the PCC and ACC (right [R] PCC: b = −0.05; SE = 0.02; *P* = .02; left [L] PCC: b = −0.04; SE = 0.02; *P* = .04; L cACC: b = −0.07; SE = 0.02; *P* = .002; L rACC: b = −0.04; SE = 0.02; *P* = .04), while higher UCH-L1 was associated with smaller left amygdala volume (b = −0.05; SE = 0.02; *P* = .02) (Table 2). Only the association between t-tau and L cACC volume passed multiple comparison correction.

**Table 2.** Associations Between Plasma Biomarker Concentration and Brain Volume^a^

Brain region	GFAP				NfL				T-tau				UCH-L1			
	b (SE)	*t*	*P* value	Partial *R*^2^	b (SE)	*t*	*P* value	Partial *R*^2^	b (SE)	*t*	*P* value	Partial *R*^2^	b (SE)	*t*	*P* value	Partial *R*^2^
R rostral ACC	−0.02 (0.06)	−0.36	.72	0.0002	−0.07 (0.05)	−1.28	.20	0.002	0.02 (0.02)	0.82	.41	0.001	0.01 (0.02)	0.60	.55	0.001
L rostral ACC	0.06 (0.05)	1.17	.24	0.002	−0.05 (0.05)	−1.14	.25	0.002	−0.04 (0.02)^b^	−2.03	.04	0.01	−0.02 (0.02)	−0.74	.46	0.001
R caudal ACC	−0.03 (0.06)	−0.47	.64	0.0003	0.05 (0.06)	0.85	.39	0.001	−0.04 (0.02)	−1.74	.08	0.004	−0.02 (0.02)	−0.89	.37	0.001
L caudal ACC	−0.04 (0.06)	−0.57	.57	0.0005	−0.02 (0.06)	−0.29	.77	0.0001	−0.07 (0.02)^b^^,^^c^	−3.10	.002	0.01	−0.03 (0.02)	−1.01	.31	0.001
R MTG	0.06 (0.05)	1.23	.22	0.002	0.05 (0.05)	1.00	.32	0.001	−0.001 (0.02)	−0.06	.96	0.000004	0.02 (0.02)	1.26	.21	0.002
L MTG	0.05 (0.05)	0.87	.39	0.001	0.05 (0.05)	0.99	.32	0.001	0.02 (0.02)	0.90	.37	0.001	0.03 (0.02)	1.33	.18	0.003
R insula	0.01 (0.05)	0.20	.84	0.0001	−0.02 (0.05)	−0.53	.60	0.0004	−0.02 (0.02)	−0.90	.37	0.001	−0.03 (0.02)	−1.53	.13	0.003
L insula	−0.02 (0.05)	−0.41	.68	0.0002	−0.02 (0.05)	−0.34	.73	0.0002	−0.04 (0.02)	−1.81	.07	0.005	−0.02 (0.02)	−1.11	.27	0.002
R amygdala	0.03 (0.06)	0.47	.64	0.0003	−0.03 (0.05)	−0.68	.50	0.001	−0.02 (0.02)	−0.94	.35	0.001	−0.04 (0.02)	−1.81	.07	0.005
L amygdala	0.09 (0.06)	1.59	.11	0.004	0.05 (0.05)	0.98	.33	0.001	−0.04 (0.02)	−1.78	.08	0.005	−0.05 (0.02)^b^	−2.32	.02	0.01
R hippocampus	0.07 (0.05)	1.27	.20	0.002	−0.02 (0.05)	−0.46	.65	0.0003	0.003 (0.02)	0.12	.90	0.00002	−0.03 (0.02)	−1.24	.21	0.002
L hippocampus	0.04 (0.06)	0.70	.49	0.001	−0.02 (0.05)	−0.39	.70	0.0002	−0.02 (0.02)	−1.17	.24	0.002	−0.03 (0.02)	−1.53	.13	0.003
R PHG	−0.003 (0.07)	−0.05	.96	0.000003	−0.02 (0.06)	−0.28	.78	0.0001	−0.02 (0.02)	−1.00	.32	0.001	−0.01 (0.03)	−0.45	.66	0.0003
L PHG	0.10 (0.07)	1.57	.12	0.004	−0.04 (0.06)	−0.70	.48	0.001	−0.02 (0.02)	−0.80	.43	0.001	−0.05 (0.03)	−1.94	.05	0.01
R PCC	0.11 (0.06)	1.88	.06	0.01	0.03 (0.05)	0.57	.57	0.0005	−0.05 (0.02)^b^	−2.25	.02	0.01	−0.04 (0.02)	−1.63	.10	0.004
L PCC	−0.07 (0.06)	−1.29	.20	0.002	−0.04 (0.05)	−0.80	.43	0.001	−0.04 (0.02)^b^	−2.01	.04	0.01	0.004 (0.02)	0.21	.84	0.0001
R IPL	0.06 (0.05)	1.05	.30	0.002	0.06 (0.05)	1.32	.19	0.003	−0.01 (0.02)	−0.48	.63	0.0003	0.02 (0.02)	0.98	.33	0.001
L IPL	0.09 (0.06)	1.62	.11	0.004	0.04 (0.05)	0.81	.42	0.001	−0.01 (0.02)	−0.50	.62	0.0004	−0.0005 (0.02)	−0.02	.98	0.000001

Abbreviations: ACC, anterior cingulate cortex; GFAP, glial fibrillary acidic protein; IPL, inferior parietal lobule; L, left; MTG, middle temporal gyrus; NfL, neurofilament light chain; PCC, posterior cingulate cortex; PHG, parahippocampal gyrus; R, right; t-tau, total tau; UCH-L1, ubiquitin C-terminal hydrolase L1.

^a^ Table includes results from linear regression analyses that examined the association between plasma biomarkers (log2 transformed) and brain volume (*z* scored). The following covariates were included in all models: age, race, education, sex, time since mild traumatic brain injury, and total intracranial volume. Partial *R*^2^ values reflect biomarker association with brain volume, among 698 participants.

^b^ Significant before false discovery rate correction.

^c^ Remains significant after false discovery rate correction.

### Plasma Biomarkers, mTBI, and Brain Volume

Among the same 698 SMVs, we examined whether blast-related and combat-related mTBIs moderated the association between plasma biomarker concentration and brain volume at enrollment. No significant associations were observed among those with 1 combat- or blast-related mTBI, but they were found for those with no or 2 combat- or blast-related mTBIs. None of these associations passed multiple comparison correction.

#### Combat-Related mTBI

**Figure 2.**
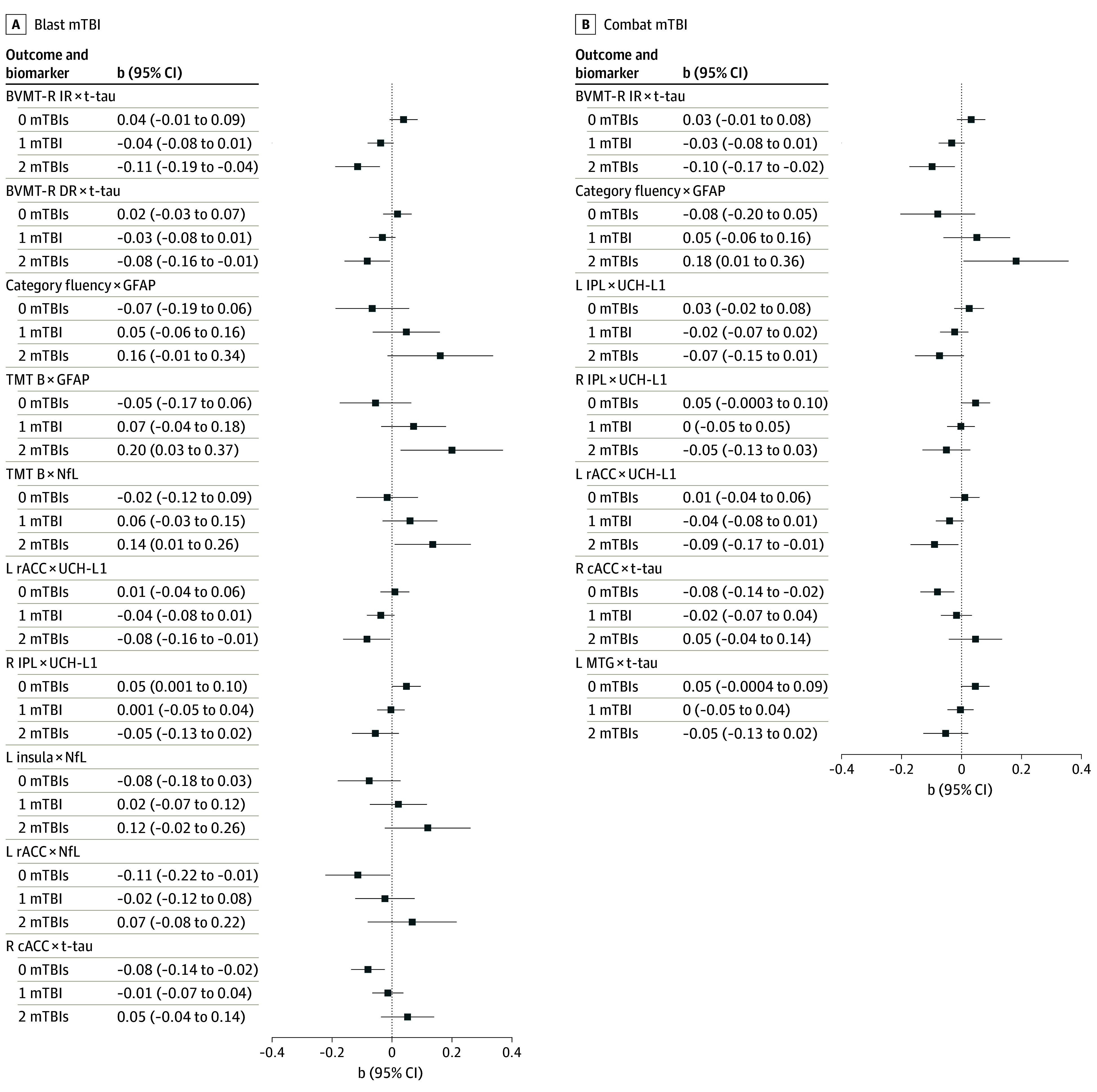
Follow-Up Simple Slopes Analyses of Interaction Between Biomarkers, Brian Volume, and Cognition for Blast- and Combat-Related Mild Traumatic Brain Injury (mTBI) Results from the follow-up simple slopes analyses of significant plasma biomarker × mTBI interactions on brain volume and cognition. BVMT-R DR indicates Brief Visuospatial Memory Test–Revised delayed recall; BVMT-R IR, Brief Visuospatial Memory Test–Revised immediate recall; cACC, caudal anterior cingulate cortex; GFAP, glial fibrillary acidic protein; IPL, inferior parietal lobule; L, left; MTG, middle temporal gyrus; NfL, neurofilament light chain; R, right; rACC, rostral anterior cingulate cortex; TMT B, Trail Making Test B; t-tau, total tau; and UCH-L1, ubiquitin C-terminal hydrolase L1.

Combat-related mTBI moderated the association between t-tau (R cACC: b = 0.06; SE = 0.03; *P* = .02; L MTG: b = −0.05; SE = 0.02; *P* = .03), UCH-L1 (L rACC: b = −0.05; SE = 0.02; *P* = .04; R IPL: b = −0.05; SE = 0.02; *P* = .04; L IPL: b = −0.05; SE = 0.02; *P* = .045) and brain volume (eTable 2 in Supplement 1). Specifically, among those with no combat-related mTBIs, higher t-tau was associated with smaller ACC volume (Figure 2; eTable 3 in Supplement 1). Among those with 2 mTBIs, higher UCH-L1 was associated with smaller ACC volume (b = −0.09; 95% CI, −0.17 to −0.01; *P* = .03) (Figure 2; eTable 3 in Supplement 1).

#### Blast-Related mTBI

Blast-related mTBI moderated the association between NfL (L rACC: b = 0.09; SE = 0.04; *P* = .03; L insula: b = 0.10; SE = 0.04; *P* = .02), t-tau (R cACC: b = 0.07; SE = 0.03; *P* = .01), and UCH-L1 (L rACC: b = −0.05; SE = 0.02; *P* = .047; R IPL: b = −0.05; SE = 0.02; *P* = .03) and brain volume (eTable 4 in Supplement 1). Among those with no blast-related mTBIs, higher t-tau and higher NfL were associated with smaller ACC volume, while higher UCH-L1 was associated with larger IPL volume (Figure 2; eTable 3 in Supplement 1). Among those with 2 blast-related mTBIs, higher UCH-L1 was associated with smaller ACC volume (b = −0.08; 95% CI, −0.16 to −0.01; *P* = .04) (Figure 2; eTable 3 in Supplement 1).

### Plasma Biomarkers and Cognition

Among all participants, we examined whether plasma biomarkers were associated with cognitive performance at enrollment. One participant was missing UCH-L1 concentration; therefore, these analyses were completed among 1159 SMVs. Higher GFAP was associated with poorer visual learning (b = −0.11; SE = 0.05; *P* = .03) and higher t-tau was associated with poorer category fluency performance (b = −0.04; SE = 0.02; *P* = .047) (Table 3). No associations passed multiple comparison correction. No other associations between plasma biomarker concentration and cognitive performance were observed.

**Table 3.** Associations Between Plasma Biomarker Concentrations and Cognitive Performance^a^

Performance measure	GFAP				NfL				T-tau				UCH-L1			
	b (SE)	*t*	*P* value	Partial *R*^2^	b (SE)	*t*	*P* value	Partial *R*^2^	b (SE)	*t*	*P* value	Partial *R*^2^	b (SE)	*t*	*P* value	Partial *R*^2^
BVMT-R IR	−0.11 (0.05)^b^	−2.22	.03	0.004	0.02 (0.04)	0.48	.63	0.0002	−0.01 (0.02)	−0.29	.77	0.0001	−0.01 (0.02)	−0.61	.54	0.0002
BVMT-R DR	−0.09 (0.05)	−1.68	.09	0.002	−0.01 (0.04)	−0.29	.77	0.0001	−0.01 (0.02)	−0.54	.59	0.0003	0.01 (0.02)	0.42	.68	0.0002
CVLT-II IR	−0.04 (0.05)	−0.68	.50	0.0004	−0.001 (0.05)	−0.03	.98	0.000001	0.01 (0.02)	0.31	.76	0.0001	−0.01 (0.02)	−0.75	.45	0.0004
CVLT-II LDFR	−0.02 (0.05)	−0.31	.76	0.0001	0.04 (0.05)	0.78	.43	0.001	0.02 (0.02)	1.14	.25	0.001	0.005 (0.02)	0.23	.82	−0.0001
Letter fluency	−0.02 (0.05)	−0.39	.70	0.0001	−0.04 (0.05)	−0.93	.35	0.001	−0.01 (0.02)	−0.58	.56	0.0003	0.03 (0.02)	1.36	.18	0.001
Category fluency	0.01 (0.05)	0.10	.92	0.00001	−0.01 (0.05)	−0.17	.87	0.00002	−0.04 (0.02)^b^	−1.99	.047	0.003	0.01 (0.02)	0.35	.73	0.0002
TMT A	0.06 (0.05)	1.22	.22	0.001	0.03 (0.05)	0.57	.57	0.0003	0.03 (0.02)	1.27	.20	0.001	0.02 (0.02)	0.87	.38	0.001
TMT B	0.02 (0.05)	0.40	.69	0.0001	0.04 (0.05)	0.78	.44	0.001	0.02 (0.02)	0.76	.45	0.001	−0.004 (0.02)	−0.21	.83	0.00003
Digit span total	0.01 (0.05)	0.10	.92	0.00001	−0.05 (0.05)	−1.10	.27	0.001	0.01 (0.02)	0.71	.48	0.0004	0.02 (0.02)	0.97	.33	0.001
Symbol Search total	0.06 (0.05)	1.13	.26	0.001	0.07 (0.04)	1.63	.10	0.002	−0.03 (0.02)	−1.48	.14	0.002	0.01 (0.02)	0.46	.64	0.0002
Coding total	0.05 (0.05)	0.90	.37	0.001	0.005 (0.04)	0.10	.92	0.00001	−0.03 (0.02)	−1.49	.14	0.002	0.005 (0.02)	0.25	.80	0.0001

Abbreviations: BVMT-R DR, Brief Visuospatial Memory Test–Revised delayed recall; BVMT-R IR, Brief Visuospatial Memory Test–Revised immediate recall; CVLT-II IR, California Verbal Learning Test-II immediate recall; CVLT-II LDFR, California Verbal Learning Test-II long delay free recall; GFAP, glial fibrillary acidic protein; NfL, neurofilament light chain; TMT, Trail Making Test; t-tau, total tau; UCH-L1, ubiquitin C-terminal hydrolase L1.

^a^ Analyses were conducted among 1160 participants for GFAP, NfL, and t-tau, and among 1159 for UCH-L1. Table includes results from linear regression analyses that examined the association between plasma biomarkers (log2 transformed) and cognitive performance (*z* scored). The following covariates were included in all models: age, race, education, sex, and time since mild traumatic brain injury. No association passed multiple comparison correction. Partial *R*^2^ values reflect biomarker association with cognitive performance.

^b^ Significant before false discovery rate correction.

### Plasma Biomarkers, mTBI, and Cognition

Next, we examined whether mTBI (both combat- and blast-related) moderated the association between plasma biomarker concentration and cognitive performance at enrollment.

#### Combat-Related TBI

Combat-related mTBI moderated the association between GFAP and category fluency (b = 0.13; SE = 0.05; *P* = .01) and between t-tau and immediate visual recall (b = −0.07; SE = 0.02; *P* = .01) (eTable 5 in Supplement 1). Among those with 2 combat-related mTBIs, higher t-tau was associated with poorer performance on visual immediate recall, while higher GFAP was associated with better performance on category fluency (Figure 2; and eTable 6 in Supplement 1). No associations passed multiple comparison correction.

#### Blast-Related TBI

Blast-related mTBI moderated the association between GFAP and category fluency (b = 0.11; SE = 0.05; *P* = .03) and TMT B (b = 0.13; SE = 0.05; *P* = .01); between NfL and TMT B (b = 0.08; SE = 0.04; *P* = .04); and t-tau and immediate (b = −0.08; SE = 0.02; *P* = .001) and delayed (b = −0.05; SE = 0.02; *P* = .03) visual recall (eTable 7 in Supplement 1). Among those with 2 blast-related mTBIs, higher t-tau was associated with poorer performance on immediate and delayed visual recall (eg, performance on the Brief Visuospatial Memory Test–Revised, immediate recall: b = −0.11; 95% CI, −0.19 to −0.04; *P* = .003; delayed recall: b = −0.08; 95% CI, −0.16 to −0.01; *P* = .03), and higher GFAP and NfL were associated with poorer TMT B performance (GFAP: b = 0.20; 95% CI, 0.03 to 0.37; *P* = .02; NfL: b = 0.14; 95% CI, 0.01 to 0.26; *P* = .04) (Figure 2; eTable 6 in Supplement 1). Only the association between t-tau, blast-related mTBI, and immediate visual recall passed multiple comparison correction.

### Plasma Biomarkers and Self-Reported Functioning–Cognitive Performance

Among 940 SMVs, higher GFAP was associated with the high self-reported functioning–high cognitive performance (odds ratio [OR], 1.95; 95% CI, 1.13-3.34) and moderate self-reported functioning–high cognitive performance (OR, 1.80; 95% CI, 1.11-2.91) groups compared with the low self-reported functioning–low cognitive performance group (after Bonferroni correction), which was unexpected (eTable 8 and eFigure 2 in Supplement 1).

### Mediation Analyses

Mediation analyses were conducted among 698 SMVs. No significant indirect effects were observed (eFigure 3 in Supplement 1).

## Discussion

In the present study we examined whether combat- and blast-related mTBI moderated the association between plasma biomarker concentration, brain volume, and cognitive performance. We found that higher concentrations of UCH-L1, t-tau, and NfL were associated with smaller ACC volumes for those with both no and 2 mTBIs but not 1 mTBI. Among participants with a greater number of mTBIs, higher t-tau was associated with poorer visual learning and memory performance, and higher GFAP and NfL were associated with poorer executive functioning performance. While several unadjusted associations were observed, few passed multiple comparison correction and thus should be interpreted with caution. Overall, findings suggest that plasma biomarkers of neuronal injury assessed in the chronic phase of mTBI are associated with brain structure and cognitive performance, and these associations vary based on the number of mTBIs.

Higher t-tau was associated with smaller ACC and PCC volumes. Prior studies are mixed, with some demonstrating that t-tau varies with brain structure,^12,36^ while another reported no association.^37^ These varied findings may be due to the mixed sensitivity of t-tau, as it has been found not to differ between TBI and control groups.^17,19,37^ T-tau is important for microtubule stability, which impacts cell shape and axonal transport. When neurons are damaged, tau detaches from microtubules and eventually forms neurofibrillary tangles.^38^ However, plasma t-tau has been found to only partially reflect brain pathology^36^ and shows weaker associations with central nervous system (CNS) function compared with phosphorylated tau (pTau).^39^ Thus, the mixed findings for t-tau may be expected. Prior studies have also shown that mTBI relates to declines in ACC volumes.^1^ The ACC is important for top-downregulation of emotions and modulating autonomic activity in response to stress.^40,41,42^ The relationship between t-tau and ACC volumes may underlie the development of posttraumatic stress disorder among those with repetitive TBIs compared with those with fewer TBIs.^43^ However, in the present study, the negative association between t-tau and ACC volumes was found primarily in those with no mTBIs rather than those with 1 or 2 mTBIs. This was somewhat unexpected, as we would expect those with greater neurological insult to exhibit a similar association. Among those with 2 mTBIs, there was an inverse association between UCH-L1 and ACC volumes. UCH-L1 is important for maintaining axonal function after brain injury, and higher concentrations may reflect increased BBB permeability or ongoing damage to neurons.^44,45,46^ After TBI, UCH-L1 increases and incrementally rises with TBI severity,^8,47^ and it distinguishes those with mTBI from trauma controls.^8^ Although higher concentrations of UCH-L1 were associated with smaller ACC volumes in those with 2 mTBIs, it was also unexpectedly associated with larger IPL volumes in those with no mTBIs. This may suggest a differential effect of UCH-L1 on the brain contingent upon neurological events. Although biomarker-mTBI-brain volume associations did not pass multiple correction and should be interpreted with caution, overall, results may be interpreted preliminarily to suggest that plasma markers of neuronal damage assessed several years after injury may be useful biomarkers of brain structure in mTBI. This is important as the effects of mTBI on the brain are typically not evident on traditional neuroimaging modalities (ie, computed tomography).

We also found that among those with 2 mTBIs, higher t-tau was associated with poorer performance on visual learning and memory. Prior studies have found that higher t-tau is associated with worse performance on immediate verbal memory,^37^ and higher pTau relates to poorer visual memory.^48^ Tau is important for both intracellular and extracellular transport within and to other neurons, and thus, dysregulation may impede learning and memory. However, we did observe some contradictory findings similar to other studies.^16,17,30^ Specifically, among those with 2 mTBIs, higher concentrations of GFAP were associated with better performance on category fluency. However, this association was not significant after multiple comparison correction. Further, in exploratory analyses using previously established profiles of cognitive performance and psychiatric functioning,^49^ lower GFAP was associated with the low self-reported functioning/low cognitive performance group, while elevated GFAP was associated with the high self-reported functioning/high cognitive performance group. This is unexpected given that we expected higher GFAP to be associated with worse cognitive performance and worse emotion-related functioning, similar to other studies.^10,50^ While some findings were contradictory, overall, results suggest that among those exposed to repetitive mTBIs, plasma biomarkers of neuronal injury may be associated with poorer cognitive performance. Of note, most associations between biomarkers and cognition did not pass multiple comparison correction and thus should be interpreted with caution. Additional studies are necessary to clarify these associations. Secondary analyses also examined indirect effects among mTBI, plasma biomarkers, brain volume, and cognition. No indirect effects were present, suggesting that the hypothesized pathways were not supported.

### Strengths and Limitations

Although the present study has several strengths, including its large sample size, comprehensive TBI interview and neuropsychological assessment, and high throughput methods to assess biomarker concentrations, it is not without limitations. First, the biomarkers included in the present study typically show the most robust associations when assessed acutely after injury,^51^ and results are mixed when biomarkers are assessed chronically.^17,52^ Second, while we showed several associations between plasma biomarkers, mTBI, brain volume, and cognitive performance, most comparisons did not pass multiple comparison correction. Further, interaction terms in particular may be vulnerable to type I error due to smaller cell counts. Therefore, replication in larger studies is important to determine whether interactions are robust. Next, many studies show that demographic factors, peripheral disease, and time since injury influence plasma biomarker concentrations.^21,53,54,55^ Although we included demographic characteristics and time since injury as covariates, other factors such as peripheral disease (eg, hypertension, diabetes) impact biomarker concentrations and thresholds in disease prediction. Further, there is a high incidence of substance use (primarily alcohol) among SMVs^56,57^ which may impact brain structure and should be considered in future studies assessing longitudinal brain changes. Additionally, we used t-tau and did not include measures of pTau, which has been shown to better reflect CNS pathology and more accurately differentiate individuals with TBI from controls.^58^ Next, the analytic sample for plasma biomarker–brain volume analyses was significantly smaller than the full analytic sample. Therefore, there is a possibility of selection bias of participants who had available MRI data vs those who did not. Finally, the present study examined cross-sectional associations between mTBI, plasma biomarker concentration, brain volume, and cognition. Future studies should employ longitudinal designs to determine whether plasma biomarkers are associated with trajectories of brain volume loss and cognitive decline as a function of mTBI.

## Conclusions

In this cross-sectional study of 1160 SMVs, blood-based biomarkers of neuronal injury assessed during the chronic phase of mTBI were associated with brain structure and cognitive performance. Given that few associations passed multiple comparison correction, the present results remain exploratory. However, our findings provide a foundation for future studies that may explore these associations within larger samples. Additionally, future studies should include important potential confounding variables, such as presence of mood and trauma-related disorders, physical symptoms, and substance use and history.

## Notes

Supplement 1.
eMethods.
**eTable 1.** Scan Parameters by Site **eTable 2.** Association Between Combat-Related mTBI, Plasma Biomarker Concentration, and Brain Volume **eTable 3.** Follow-Up Simple Slopes Analyses for Brain Volume **eTable 4.** Association Between Blast-Related mTBI, Plasma Biomarker Concentration, and Brain Volume **eTable 5.** Association Between Combat-Related mTBI, Plasma Biomarker Concentration, and Cognitive Performance **eTable 6.** Follow-Up Simple Slopes Analyses for Cognitive Performance **eTable 7.** Association Between Blast-Related mTBI, Plasma Biomarker Concentration, and Cognitive Performance **eTable 8.** Association Between Plasma Biomarker Concentration and Self-Reported Functioning–Cognition Profiles **eFigure 1.** Study Timeline and Exclusion Flowchart **eFigure 2.** GFAP by Self-Reported Functioning/Cognitive Performance Profile **eFigure 3.** Serial Mediation Analyses
eReferences.

Supplement 2.
Data Sharing Statement
