# Supplementary material for: Plasma Biomarkers, Brain Volume, and Cognitive Performance in Service Members and Veterans With mTBI: A LIMBIC-CENC Study
Source: JAMA Netw Open. 2026 Feb 25;9(2):e2559596. doi: 10.1001/jamanetworkopen.2025.59596 (PMC12936882; doi:10.1001/jamanetworkopen.2025.59596)
Supplement: Supplement 1. — eMethods. eTable 1. Scan Parameters by Site eTable 2. Association Between Combat-Related mTBI, Plasma Biomarker Concentration, and Brain Volume eTable 3. Follow-Up Simple Slopes Analyses for Brain Volume eTable 4. Association Between Blast-Related mTBI, Plasma Biomarker Concentration, and Brain Volume eTable 5. Association Between Combat-Related mTBI, Plasma Biomarker Concentration, and Cognitive Performance eTable 6. Follow-Up Simple Slopes Analyses for Cognitive Performance eTable 7. Association Between Blast-Related mTBI, Plasma Biomarker Concentration, and Cognitive Performance eTable 8. Association Between Plasma Biomarker Concentration and Self-Reported Functioning–Cognition Profiles eFigure 1. Study Timeline and Exclusion Flowchart eFigure 2. GFAP by Self-Reported Functioning/Cognitive Performance Profile eFigure 3. Serial Mediation Analyses eReferences. [file jamanetwopen-e2559596-s001.pdf]

Supplemental Online Content

Dark HE, Kenney K, de Souza NL, et al. Plasma biomarkers, brain volume, and cognitive performance in service members and veterans with mTBI. *JAMA Netw Open.* 2026;9(2):e2559596. doi:10.1001/jamanetworkopen.2025.59596

eMethods.

eTable 1. Scan Parameters by Site

eTable 2. Association Between Combat-Related mTBI, Plasma Biomarker Concentration, and Brain Volume

eTable 3. Follow-Up Simple Slopes Analyses for Brain Volume

eTable 4. Association Between Blast-Related mTBI, Plasma Biomarker Concentration, and Brain Volume

eTable 5. Association Between Combat-Related mTBI, Plasma Biomarker Concentration, and Cognitive Performance

eTable 6. Follow-Up Simple Slopes Analyses for Cognitive Performance

eTable 7. Association Between Blast-Related mTBI, Plasma Biomarker Concentration, and Cognitive Performance

eTable 8. Association Between Plasma Biomarker Concentration and Self-Reported Functioning–Cognition Profiles

eFigure 1. Study Timeline and Exclusion Flowchart

eFigure 2. GFAP by Self-Reported Functioning/Cognitive Performance Profile

eFigure 3. Serial Mediation Analyses

eReferences.

This supplemental material has been provided by the authors to give readers additional information about their work.

© 2026 Dark HE et al. *JAMA Network Open*.

eMethods

Participants
Participants in the present sample were selected if they had blood samples, magnetic resonance imaging (MRI), and cognitive data collected at enrollment. In the present study, participants were excluded from analyses for missing covariate data or invalid neuropsychological profile data as defined by invalid profile on the Medical Symptom Validity test (MSVT)[3] (**eFigure 1**). All study procedures were approved by respective LIMBIC-CENC enrollment sites and all participants provided informed consent.

Plasma biomarkers
Samples were run using the Simoa HD-X instrument. Samples were excluded if the coefficient of variation (CV) was ≥0.20. Intrassay CVs were all <10%.

TBI assessment
On interview, participants were first asked to recall all injuries over the lifetime. Loss of consciousness (LOC), duration of LOC, PTA, and age corresponding to each injury reported in step 1 were obtained. Participants were then asked whether they experienced a period of repeated head injuries, including LOC, PTA, the most severe effects, and initial age and ending of period of repeated injuries. Events were classified as mild TBI vs not mild TBI based on the Department of Veterans Affairs and Department of Defense practice guidelines[4]. mTBIs were further classified based on whether they occurred during combat, and whether they were blast related. Participants' first, most recent, and index ('worst' combat mTBI) mTBIs were also assessed. They were excluded if they endorsed moderate or severe TBI. Combat-related, and blast-related mTBIs were used as moderator variables for the present study.

Neuropsychological assessment
The MSVT cutoff for invalid response profiles was :<85% on the immediate or delayed recall subtests. Attention/working memory was assessed using the digit span total score from the Wechsler Adult Intelligence Scale 4th edition (WAIS-IV)[5]. Processing speed was assessed using the Trail Making Test Part A[6] and Symbol Search and Coding subtests from the WAIS-IV[5]. Language and executive functioning were assessed using the Delis–Kaplan Executive Function System Letter and Category fluency[7] and Trail Making Test Part B[6]. Visual learning and memory were assessed using the Brief Visuospatial Memory Test-Revised (BVMT-R) immediate recall and delayed recall trials[8], respectively. Finally, verbal learning and memory were assessed using the California Verbal Learning Test Second Edition (CVLT-II) Trails 1-5 total score and long delay free recall total score[9], respectively.

Self-reported functioning and cognitive performance profiles
In a previous study[10] using a latent profile analysis, 6 profiles of self-reported functioning/cognitive performance were generated using psychological symptom reports and cognitive performance data collected during enrollment for a subset of the current sample[10]. Previously established groups included: High self-reported functioning/High cognitive performance (HS/HC), High self-reported functioning/Low cognitive performance (HS/LC), Moderate self-reported functioning/High cognitive performance (MS/HC), Moderate self-reported functioning/Low cognitive performance (MS/LC), Low self-reported functioning/High cognitive performance (LS/HC), Low self-reported functioning/Low cognitive performance (LS/LC). Full description of self-reported symptom and cognitive performance measures and methods for generating profiles have been previously described[10].

MRI acquisition
T1-weighted magnetization-prepared rapid gradient echo (MPRAGE) scans were collected at enrollment using a similar protocol to the Alzheimer Disease Neuroimaging Initiative network model[37]. Scan parameters per site have been previously published[23, 38].

Brain volume
Brain volume was computed by multiplying the voxel volume by the total number of voxels in each region using previously described methods[39-41]. Estimated total intracranial volume (eTIV) was estimated by FreeSurfer by computing an atlas scaling factor based on the affine transformation of each participants' image to the atlas-based template[42]. Brain regions of interest (ROIs) included the bilateral rostral anterior cingulate cortex (rACC), caudal anterior cingulate cortex (cACC), middle temporal gyrus (MTG), insula, amygdala, hippocampus, parahippocampal

© 2026 Dark HE et al. *JAMA Network Open*.

gyrus (PHG), posterior cingulate cortex (PCC), and inferior parietal lobule (IPL). The 18 ROIs were selected based on their association with mTBI.

Covariates

Time since last mTBI was initially a continuous variable (in years); however, to include those with 0 mTBIs in analyses, time since last mTBI was grouped (No mTBI/NA=0, <1 year=1, ≥1 and <3 years=2, ≥3 and <7 years=3, ≥7 years and <10 years=4, ≥10 years=5). Time since mTBI was used since biomarker concentrations are elevated closer to injury.

Statistical analyses

To further examine two-way interactions, follow-up simple slopes analyses were completed by examining the relationship between plasma biomarkers and brain volume by mTBI (0, 1, and 2). Due to the sample size of participants with ≥3 mTBIs, slopes at these levels were not examined. Associations between plasma biomarkers and cognitive performance were completed in kind to plasma biomarker-brain volume analyses except eTIV was not included as a covariate in cognitive performance analyses. A false discovery rate (FDR) approach was applied to *P* values within each group of biomarker analyses. For example, for analyses that examined the relationship of GFAP to brain volume, all *P* values for each linear regression analysis relating GFAP and brain volume (18 ROIs) were entered into *p.adjust* in R to determine which remained significant after correcting for multiple comparisons ($P_{FDR}<0.05$). Each model that examined associations between plasma biomarkers and cognition was checked for linear regression assumptions. No models contained influential outliers, and the distribution of residuals were normally distributed or approaching normality. In a subgroup of participants (n=940), multinomial logistic regression analyses were completed to determine whether enrollment plasma biomarker concentration was related to previously defined self-reported functioning/cognitive performance profiles. Profiles were generated using latent profile analyses which has been previously described[10]. The LS/LC group was used as a reference and Bonferroni correction was used for pairwise comparisons. Finally, based on significant associations in *biomarker-mTBI-brain* and *biomarker-mTBI-cognition* analyses, follow-up serial mediation analyses were completed to determine whether plasma biomarker concentration (mediator 1) and brain volume (mediator 2) serially mediated the relationship between mTBI (predictor) and cognitive performance (outcome). We sought to determine the indirect effects of mTBI, plasma biomarkers, and brain volume on cognitive performance. Mediation analyses were conducted using the PROCESS macro (model 6)[11] in SPSS. Ninety-five percent confidence intervals for indirect effects were estimated using 10,000 bootstrapped samples.

© 2026 Dark HE et al. *JAMA Network Open*.

eTable 1. Scan Parameters by Site

Site	Location	Scanner	TR (ms)	TE (ms)	Voxel size (mm)	Matrix	Slices
1	Richmond, VA	Philips Ingenia	6.78	3.157	1.0 × 1.0 × 1.2	256 × 256	176
2	Houston, TX	Siemens TrioTim	2300	2.96	1.0 × 1.0 × 1.2	240 × 256	176
4	San Antonio, TX	Siemens Verio/Skyra Fit	2300	2.98	1.0 × 1.0 × 1.2	240 × 256	176
6	Portland, OR	Philips Achieva	6.76	3.145	1.0 × 1.0 × 1.2	256 × 256	170
7	Minneapolis, MN	Siemens Prisma	2400	2.24	0.8 × 0.8 × 0.8	300 × 320	208
8	Boston, MA	Siemens Prisma	2400	2.24	0.8 × 0.8 × 0.8	300 × 320	208

© 2026 Dark HE et al. *JAMA Network Open*.

eTable 2. Association Between Combat-Related mTBI, Plasma Biomarker Concentration, and Brain Volume

	GFAP × Combat mTBI					NfL × Combat mTBI				
	b	SE	t	*P*	Partial R^2	b	SE	t	*P*	Partial R^2
R Rostral ACC	0.04	0.06	0.65	0.51	0.001	0.05	0.05	1.16	0.25	0.002
L Rostral ACC	0.01	0.06	0.10	0.92	0.00001	0.06	0.04	1.29	0.20	0.002
R Caudal ACC	-0.03	0.07	-0.47	0.64	0.0003	0.004	0.05	0.08	0.94	0.00001
L Caudal ACC	-0.002	0.07	-0.03	0.98	0.000001	0.03	0.05	0.59	0.56	0.0005
R MTG	-0.02	0.06	-0.44	0.66	0.0003	0.02	0.04	0.42	0.67	0.0003
L MTG	0.03	0.06	0.53	0.60	0.0004	0.01	0.04	0.20	0.84	0.0001
R Insula	-0.05	0.06	-0.83	0.40	0.001	0.02	0.04	0.52	0.60	0.0004
L Insula	0.01	0.06	0.23	0.81	0.0001	0.08	0.04	1.86	0.06	0.01
R amygdala	0.06	0.06	1.01	0.31	0.001	0.04	0.04	0.81	0.42	0.001
L amygdala	0.04	0.06	0.64	0.52	0.001	0.005	0.05	0.10	0.92	0.00001
R hippocampus	0.02	0.06	0.30	0.76	0.0001	0.01	0.04	0.21	0.83	0.0001
L hippocampus	0.09	0.06	1.49	0.14	0.003	0.04	0.05	0.93	0.35	0.001
R PHG	0.04	0.07	0.54	0.59	0.0004	0.01	0.05	0.21	0.83	0.0001
L PHG	0.04	0.07	0.61	0.54	0.001	0.05	0.05	0.96	0.34	0.001
R PCC	-0.04	0.06	-0.72	0.47	0.001	0.06	0.05	1.37	0.17	0.003
L PCC	-0.05	0.06	-0.89	0.37	0.001	0.03	0.04	0.76	0.45	0.001
R IPL	-0.03	0.06	-0.45	0.65	0.0003	0.04	0.04	0.90	0.37	0.001
L IPL	0.04	0.06	0.73	0.46	0.001	0.03	0.04	0.66	0.51	0.001
	T-tau × Combat mTBI					UCH-L1 × Combat mTBI				
	b	SE	t	*P*	Partial R^2	b	SE	t	*P*	Partial R^2
R Rostral ACC	0.04	0.03	1.41	0.16	0.003	-0.02	0.03	-0.64	0.52	0.001
L Rostral ACC	0.01	0.02	0.61	0.54	0.001	**-0.05**	**0.02**	**-2.09**	**0.04**	**0.01**
R Caudal ACC	**0.06**	**0.03**	**2.35**	**0.02**	**0.01**	0.01	0.03	0.29	0.77	0.0001
L Caudal ACC	-0.04	0.03	-1.55	0.12	0.003	-0.03	0.03	-0.94	0.35	0.001
R MTG	-0.03	0.02	-1.28	0.20	0.002	-0.03	0.02	-1.24	0.21	0.002
L MTG	**-0.05**	**0.02**	**-2.15**	**0.03**	**0.01**	-0.01	0.02	-0.24	0.81	0.0001
R Insula	0.01	0.02	0.46	0.64	0.0003	0.02	0.02	0.99	0.32	0.001
L Insula	-0.01	0.02	-0.30	0.76	0.0001	0.01	0.02	0.39	0.69	0.0002
R amygdala	0.03	0.02	1.21	0.23	0.002	0.003	0.02	0.12	0.91	0.00002
L amygdala	-0.01	0.02	-0.52	0.61	0.0004	-0.01	0.03	-0.29	0.77	0.0001
R hippocampus	-0.02	0.02	-0.97	0.33	0.001	0.02	0.02	0.83	0.41	0.001
L hippocampus	-0.03	0.02	-1.09	0.28	0.002	0.03	0.03	1.17	0.24	0.002
R PHG	-0.03	0.03	-1.16	0.25	0.002	0.05	0.03	1.54	0.13	0.003
L PHG	-0.004	0.03	-0.14	0.89	0.00003	0.01	0.03	0.43	0.67	0.0003
R PCC	0.02	0.02	0.82	0.41	0.001	-0.003	0.03	-0.12	0.90	0.00002
L PCC	-0.02	0.02	-0.83	0.41	0.001	-0.04	0.02	-1.60	0.11	0.004
R IPL	-0.03	0.02	-1.14	0.25	0.002	**-0.05**	**0.02**	**-2.02**	**0.04**	**0.01**
L IPL	-0.02	0.02	-1.00	0.32	0.001	**-0.05**	**0.02**	**-2.01**	**0.045**	**0.01**

Note. n=698. Table includes results from linear regression analyses that examined whether number of combat-related mTBIs moderated the relationship between plasma biomarkers (log2 transformed) and brain volume (z-scored). The following covariates were included in all models: age, race, education, sex, time since mTBI, total intracranial volume. No associations pass multiple comparison correction. Partial R^2 values reflect *biomarker* × Combat mTBI association with brain volume. *Abbreviations*: PCC: posterior cingulate cortex, ACC: anterior cingulate cortex, MTG: middle temporal gyrus, IPL: inferior parietal lobule, PHG: parahippocampal gyrus, GFAP: glial fibrillary acidic protein, NfL: Neurofilament light chain, T-tau: total tau, UCH-L1: Ubiquitin C-terminal hydrolase L1.

© 2026 Dark HE et al. *JAMA Network Open*.

eTable 3. Follow-Up Simple Slopes Analyses for Brain Volume

		0 mTBIs				1 mTBI				2 mTBIs			
		b	SE	t	*P*	b	SE	t	*P*	b	SE	t	*P*
**L rACC**	**UCHL1 x Blast mTBI**	0.01	0.02	0.41	0.68	-0.04	0.02	-1.57	0.12	**-0.08**	**0.04**	**-2.09**	**0.04**
**L rACC**	**NfL x Blast mTBI**	**-0.11**	**0.06**	**-2.05**	**0.04**	-0.02	0.05	-0.45	0.65	0.07	0.08	0.90	0.37
**L rACC**	**UCHL1 x Combat mTBI**	0.01	0.02	0.50	0.62	-0.04	0.02	-1.63	0.10	**-0.09**	**0.04**	**-2.18**	**0.03**
**R cACC**	**Tau x Blast mTBI**	**-0.08**	**0.03**	**-2.80**	**0.01**	-0.01	0.03	-0.51	0.61	0.05	0.05	1.17	0.24
**R cACC**	**Tau x Combat mTBI**	**-0.08**	**0.03**	**-2.75**	**0.01**	-0.02	0.03	-0.59	0.55	0.05	0.05	1.06	0.29
**R IPL**	**UCHL1 x Blast mTBI**	**0.05**	**0.02**	**2.01**	**0.045**	0.00	0.02	-0.12	0.90	-0.05	0.04	-1.37	0.17
L MTG	Tau x Combat mTBI	0.05	0.02	1.94	0.05	-0.002	0.02	-0.09	0.93	-0.05	0.04	-1.35	0.18
L insula	NfL x Blast mTBI	-0.08	0.05	-1.42	0.16	0.02	0.05	0.45	0.65	0.12	0.07	1.64	0.10
L IPL	UCHL1 x Combat mTBI	0.03	0.03	1.06	0.29	-0.02	0.02	-0.95	0.34	-0.07	0.04	-1.74	0.08
R IPL	UCHL1 x Combat mTBI	0.05	0.02	1.95	0.05	0.00	0.02	-0.03	0.98	-0.05	0.04	-1.22	0.22

Note. n=698. Table includes results from the follow-up simple slopes analyses based on significant *plasma biomarker* × traumatic brain injury (TBI) interactions from the linear regression analyses in eTables 2 and 4. Follow-up analyses were completed to examine associations between plasma biomarkers and brain volume at different levels of mTBI (0, 1, or 2 mTBIs). *Abbreviations*: R: right, L; left; cACC: caudal anterior cingulate cortex; rACC: rostral anterior cingulate cortex; MTG: middle temporal gyrus; IPL: inferior parietal lobule; NfL: Neurofilament light chain, T-tau: total tau; UCH-L1: Ubiquitin C-terminal hydrolase L1.

© 2026 Dark HE et al. *JAMA Network Open*.

eTable 4. Association Between Blast-Related mTBI, Plasma Biomarker Concentration, and Brain Volume

	GFAP × Blast mTBI					NfL × Blast mTBI				
	b	SE	t	*P*	Partial R^2	b	SE	t	*P*	Partial R^2
R Rostral ACC	0.04	0.07	0.58	0.56	0.0005	0.05	0.05	1.08	0.28	0.002
L Rostral ACC	0.04	0.06	0.69	0.49	0.001	**0.09**	**0.04**	**2.12**	**0.03**	**0.01**
R Caudal ACC	-0.003	0.07	-0.05	0.96	0.000003	0.01	0.05	0.27	0.78	0.0001
L Caudal ACC	0.05	0.07	0.68	0.49	0.001	0.05	0.05	0.94	0.35	0.001
R MTG	-0.03	0.06	-0.50	0.62	0.0004	0.03	0.04	0.83	0.41	0.001
L MTG	0.03	0.06	0.46	0.65	0.0003	0.03	0.04	0.69	0.49	0.001
R Insula	-0.03	0.06	-0.58	0.56	0.0005	0.04	0.04	0.86	0.39	0.001
L Insula	0.01	0.06	0.17	0.87	0.00004	**0.10**	**0.04**	**2.37**	**0.02**	**0.01**
R amygdala	0.05	0.06	0.80	0.42	0.001	0.03	0.04	0.64	0.52	0.001
L amygdala	0.05	0.06	0.81	0.42	0.001	0.004	0.04	0.08	0.94	0.00001
R hippocampus	0.01	0.06	0.11	0.91	0.00002	-0.01	0.04	-0.13	0.90	0.00002
L hippocampus	0.09	0.06	1.50	0.13	0.003	0.02	0.04	0.47	0.64	0.0003
R PHG	0.06	0.07	0.75	0.45	0.001	0.01	0.05	0.22	0.83	0.0001
L PHG	0.05	0.07	0.66	0.51	0.001	0.05	0.05	0.96	0.34	0.001
R PCC	-0.06	0.06	-0.89	0.38	0.001	0.05	0.04	1.20	0.23	0.002
L PCC	-0.02	0.06	-0.34	0.74	0.0002	0.04	0.04	0.84	0.40	0.001
R IPL	-0.04	0.06	-0.62	0.53	0.001	0.04	0.04	0.89	0.37	0.001
L IPL	0.03	0.06	0.47	0.64	0.0003	0.03	0.04	0.77	0.44	0.001

	Tau × Blast mTBI					UCH-L1 × Blast mTBI				
	b	SE	t	*P*	Partial R^2	b	SE	t	*P*	Partial R^2
R Rostral ACC	0.04	0.02	1.53	0.13	0.003	-0.02	0.03	-0.72	0.47	0.001
L Rostral ACC	0.02	0.02	0.99	0.32	0.001	**-0.05**	**0.02**	**-1.99**	**0.047**	**0.01**
R Caudal ACC	**0.07**	**0.03**	**2.47**	**0.01**	**0.01**	0.004	0.03	0.16	0.87	0.00004
L Caudal ACC	-0.04	0.03	-1.51	0.13	0.003	-0.02	0.03	-0.89	0.37	0.001
R MTG	-0.02	0.02	-0.79	0.43	0.001	-0.03	0.02	-1.20	0.23	0.002
L MTG	-0.04	0.02	-1.86	0.06	0.005	-0.01	0.02	-0.42	0.67	0.0003
R Insula	0.004	0.02	0.19	0.85	0.0001	0.02	0.02	0.93	0.35	0.001
L Insula	-0.01	0.02	-0.44	0.66	0.0003	0.01	0.02	0.48	0.63	0.0003
R amygdala	0.03	0.02	1.47	0.14	0.003	0.02	0.02	0.85	0.39	0.001
L amygdala	-0.004	0.02	-0.17	0.86	0.00004	0.002	0.02	0.07	0.94	0.00001
R hippocampus	-0.02	0.02	-0.92	0.36	0.001	0.03	0.02	1.24	0.21	0.002
L hippocampus	-0.03	0.02	-1.11	0.27	0.002	0.04	0.02	1.46	0.14	0.003
R PHG	-0.01	0.03	-0.53	0.60	0.0004	0.05	0.03	1.87	0.06	0.01
L PHG	0.01	0.03	0.44	0.66	0.0003	0.03	0.03	0.97	0.33	0.001
R PCC	0.02	0.02	0.91	0.37	0.001	-0.001	0.02	-0.04	0.97	0.000002
L PCC	-0.02	0.02	-0.75	0.45	0.001	-0.04	0.02	-1.60	0.11	0.004
R IPL	-0.03	0.02	-1.15	0.25	0.002	**-0.05**	**0.02**	**-2.21**	**0.03**	**0.01**
L IPL	-0.02	0.02	-0.92	0.36	0.001	-0.05	0.02	-1.97	0.05	0.01

Note. n=698. Table includes results from linear regression analyses that examined whether number of blast-related mTBIs moderated the relationship between plasma biomarkers (log2 transformed) and brain volume (z-scored). The following covariates were included in all models: age, race, education, sex, time since mTBI, total intracranial volume. No associations pass multiple comparison correction. Partial R^2 values reflect *biomarker* × Blast mTBI association with brain volume. *Abbreviations*: PCC: posterior cingulate cortex, ACC: anterior cingulate cortex, MTG: middle temporal gyrus, IPL: inferior parietal lobule, PHG: parahippocampal gyrus, GFAP: glial fibrillary acidic protein, NfL: Neurofilament light chain, T-tau: total tau, UCH-L1: Ubiquitin C-terminal hydrolase L1.

© 2026 Dark HE et al. *JAMA Network Open*.

eTable 5. Association Between Combat-Related mTBI, Plasma Biomarker Concentration, and Cognitive Performance

	GFAP × Combat mTBI					NfL × Combat mTBI				
	b	SE	t	*P*	Partial R^2	b	SE	t	*P*	Partial R^2
BVMT-R IR	0.03	0.05	0.64	0.52	0.0004	0.02	0.04	0.55	0.58	0.0003
BVMT-R DR	0.001	0.05	0.01	0.99	0.0000001	-0.02	0.04	-0.54	0.59	0.0003
CVLT-II IR	0.04	0.05	0.75	0.45	0.0005	0.05	0.04	1.41	0.16	0.002
CVLT-II LDFR	0.07	0.05	1.37	0.17	0.002	0.03	0.04	0.86	0.39	0.001
Letter fluency	0.05	0.05	1.01	0.31	0.001	0.04	0.04	0.94	0.35	0.001
**Category fluency**	**0.13**	**0.05**	**2.47**	**0.01**	**0.01**	0.03	0.04	0.83	0.41	0.001
TMT A	-0.01	0.05	-0.27	0.78	0.0001	-0.03	0.04	-0.84	0.40	0.001
TMT B	0.08	0.05	1.54	0.12	0.002	0.06	0.04	1.52	0.13	0.002
Digit span total	0.01	0.05	0.28	0.78	0.0001	0.03	0.04	0.81	0.42	0.001
Symbol Search total	-0.04	0.05	-0.79	0.43	0.001	-0.03	0.04	-0.95	0.34	0.001
Coding total	-0.02	0.05	-0.46	0.64	0.0002	-0.01	0.04	-0.41	0.68	0.0001
	T-tau × Combat mTBI					UCH-L1 × Combat mTBI				
	b	SE	t	*P*	Partial R^2	b	SE	t	*P*	Partial R^2
**BVMT-R IR**	**-0.07**	**0.02**	**-2.80**	**0.01**	**0.01**	-0.02	0.02	-1.02	0.31	0.001
BVMT-R DR	-0.04	0.02	-1.72	0.08	0.003	-0.04	0.02	-1.72	0.09	0.003
CVLT-II IR	-0.03	0.02	-1.41	0.16	0.002	0.02	0.02	0.81	0.42	0.001
CVLT-II LDFR	-0.04	0.02	-1.63	0.10	0.002	0.01	0.02	0.30	0.77	0.0001
Letter fluency	-0.001	0.02	-0.03	0.98	0.000001	-0.02	0.02	-0.69	0.49	0.0004
Category fluency	0.02	0.02	0.97	0.33	0.001	0.02	0.02	1.01	0.31	0.001
TMT A	0.01	0.02	0.30	0.77	0.0001	-0.02	0.02	-0.67	0.50	0.0004
TMT B	0.02	0.02	0.82	0.41	0.001	0.003	0.02	0.15	0.88	0.00002
Digit span total	-0.004	0.02	-0.15	0.88	0.00002	0.003	0.02	0.15	0.88	0.00002
Symbol Search total	-0.01	0.02	-0.35	0.72	0.0001	0.01	0.02	0.27	0.79	0.0001
Coding total	-0.01	0.02	-0.25	0.81	0.0001	-0.02	0.02	-0.68	0.50	0.0004

Note. n=1160 (UCH-L1: n=1159). Table includes results from linear regression analyses that examined whether number of combat-related mTBIs moderated the relationship between plasma biomarkers (log2 transformed) and cognitive performance (z-scored). The following covariates were included in all models: age, race, education, sex, time since mTBI. No associations pass multiple comparison correction. Partial R^2 values reflect *biomarker* × Combat mTBI association with cognitive performance. *Abbreviations*: BVMT-R: Brief Visuospatial Memory Test–Revised, CVLT-II: California Verbal Learning Test-II, TMT: Trail making test, TBI: traumatic brain injury, GFAP: glial fibrillary acidic protein, NfL: Neurofilament light chain, T-tau: total tau, UCH-L1: Ubiquitin C-terminal hydrolase L1, IR: immediate recall, DR: delayed recall, LDFR: long delay free recall.

© 2026 Dark HE et al. *JAMA Network Open*.

eTable 6. Follow-Up Simple Slopes Analyses for Cognitive Performance

		0 mTBIs				1 mTBI				2 mTBIs			
		b	SE	t	*P*	b	SE	t	*P*	b	SE	t	*P*
**BVMT-R IR**	**T-tau × Combat mTBI**	0.03	0.02	1.35	0.18	-0.03	0.02	-1.49	0.14	**-0.10**	**0.04**	**-2.55**	**0.01**
	**T-tau × Blast mTBI**	0.04	0.02	1.64	0.10	-0.04	0.02	-1.71	0.09	**-0.11**	**0.04**	**-3.01**	**0.003**
**BVMT-R DR**	**T-tau × Blast mTBI**	0.02	0.02	0.77	0.44	-0.03	0.02	-1.43	0.15	**-0.08**	**0.04**	**-2.14**	**0.03**
**Cat. fluency**	**GFAP × Combat mTBI**	-0.08	0.06	1.24	0.21	0.05	0.06	0.91	0.36	**0.18**	**0.09**	**2.04**	**0.04**
	GFAP × Blast mTBI	-0.07	0.06	-1.04	0.30	0.05	0.06	0.84	0.40	0.16	0.09	1.80	0.07
**TMT-B**	**NfL × Blast mTBI**	-0.02	0.05	-0.31	0.76	0.06	0.05	1.29	0.20	**0.14**	**0.06**	**2.10**	**0.04**
	**GFAP × Blast mTBI**	-0.05	0.06	-0.90	0.37	0.07	0.06	1.31	0.19	**0.20**	**0.09**	**2.29**	**0.02**

Note. n=1160 (UCH-L1: n=1159). Table includes results from the follow-up simple slopes analyses based on significant *plasma biomarker* × mTBI interactions from the linear regression analyses in eTables 5 and 7. Follow-up analyses were completed to examine associations between plasma biomarkers and cognitive performance at different levels of mTBI (0, 1, or 2 mTBIs). *Abbreviations*: Cat. Fluency: Category fluency; BVMT-IR: Brief Visuospatial Memory Test–Revised Immediate Recall, BVMT-DR: Brief Visuospatial Memory Test–Revised Delayed Recall, TMT-B: Trail making test B, GFAP: glial fibrillary acidic protein, NfL: Neurofilament light chain, T-tau: total tau.

© 2026 Dark HE et al. *JAMA Network Open*.

eTable 7. Association Between Blast-Related mTBI, Plasma Biomarker Concentration, and Cognitive Performance

	GFAP × Blast mTBI					NfL × Blast mTBI				
	b	SE	t	*P*	Partial R^2	b	SE	t	*P*	Partial R^2
BVMT-R IR	0.05	0.05	0.92	0.36	0.001	0.02	0.04	0.52	0.60	0.0002
BVMT-R DR	0.01	0.05	0.16	0.87	0.00002	-0.03	0.04	-0.75	0.45	0.0005
CVLT-II IR	0.04	0.05	0.78	0.44	0.001	0.07	0.04	1.97	0.05	0.003
CVLT-II LDFR	0.07	0.05	1.28	0.20	0.001	0.05	0.04	1.32	0.19	0.002
Letter fluency	0.06	0.05	1.06	0.29	0.001	0.03	0.04	0.86	0.39	0.001
**Category fluency**	**0.11**	**0.05**	**2.16**	**0.03**	**0.004**	0.02	0.04	0.67	0.51	0.0004
TMT A	0.03	0.05	0.61	0.54	0.0003	-0.01	0.04	-0.40	0.69	0.0001
**TMT B**	**0.13**	**0.05**	**2.50**	**0.01**	**0.01**	**0.08**	**0.04**	**2.11**	**0.04**	**0.004**
Digit span total	-0.02	0.05	-0.39	0.70	0.0001	0.01	0.04	0.29	0.77	0.0001
Symbol Search total	-0.06	0.05	-1.28	0.20	0.001	-0.02	0.04	-0.45	0.65	0.0002
Coding total	-0.05	0.05	-1.09	0.27	0.001	-0.01	0.04	-0.24	0.81	0.00005
	T-tau × Blast mTBI					UCH-L1 × Blast mTBI				
	b	SE	t	*P*	Partial R^2	b	SE	t	*P*	Partial R^2
**BVMT-R IR**	**-0.08**	**0.02**	**-3.34**	**0.001***	**0.01**	-0.02	0.02	-0.94	0.35	0.001
**BVMT-R DR**	**-0.05**	**0.02**	**-2.17**	**0.03**	**0.004**	-0.04	0.02	-1.63	0.10	0.002
CVLT-II IR	-0.03	0.02	-1.27	0.20	0.001	0.03	0.02	1.20	0.23	0.001
CVLT-II LDFR	-0.04	0.02	-1.46	0.14	0.002	0.01	0.02	0.53	0.59	0.0002
Letter fluency	0.004	0.02	0.17	0.86	0.00003	-0.003	0.02	-0.12	0.91	0.00001
Category fluency	0.02	0.02	0.73	0.46	0.0005	0.03	0.02	1.27	0.20	0.001
TMT A	0.01	0.02	0.49	0.62	0.0002	-0.02	0.02	-0.82	0.41	0.001
TMT B	0.03	0.02	1.31	0.19	0.001	-0.002	0.02	-0.07	0.94	0.000004
Digit span total	-0.01	0.02	-0.48	0.63	0.0002	0.01	0.02	0.42	0.68	0.0002
Symbol Search total	-0.02	0.02	-0.79	0.43	0.001	0.01	0.02	0.51	0.61	0.0002
Coding total	-0.01	0.02	-0.48	0.63	0.0002	-0.01	0.02	-0.46	0.64	0.0002

Note. n=1160 (UCH-L1: n=1159). Table includes results from linear regression analyses that examined whether number of blast-related mTBIs moderated the relationship between plasma biomarkers (log2 transformed) and cognitive performance (z-scored). The following covariates were included in all models: age, race, education, sex, time since mTBI. *P_{FDR}<0.05. Partial R^2 values reflect *biomarker* × Blast mTBI association with cognitive performance. *Abbreviations*: BVMT-R: Brief Visuospatial Memory Test–Revised, CVLT-II: California Verbal Learning Test-II, TMT: Trail making test, TBI: traumatic brain injury, GFAP: glial fibrillary acidic protein, NfL: Neurofilament light chain, T-tau: total tau, UCH-L1: Ubiquitin C-terminal hydrolase L1, DR: delayed recall, LDFR: long delay free recall.

© 2026 Dark HE et al. *JAMA Network Open*.

eTable 8. Association Between Plasma Biomarker Concentration and Self-Reported Functioning–Cognition Profiles

	Likelihood ratio tests			Model fit	
Biomarker	Chi-sq	*P*	Nagelkerke R^2	Chi-sq	*P*
Tau(log2)	3.22	0.67	0.26	258.71	<0.001
NFL(log2)	3.76	0.59	0.26	259.25	<0.001
**GFAP(log2)**	**12.70**	**0.03**	**0.27**	**268.19**	**<0.001**
UCHL1(log2)	2.88	0.72	0.26	258.37	<0.001
			Parameter estimates		
GFAP (log2)	b	SE	*P*	OR	95% CI (LL, UL)
HS/LC	0.31	0.26	0.24	1.36	0.82, 2.28
MS/LC	0.36	0.24	0.14	1.43	0.89, 2.31
**HS/HC**	**0.67**	**0.28**	**0.02**	**1.95**	**1.13, 3.34**
LS/HC	0.01	0.27	0.96	1.01	0.60, 1.71
**MS/HC**	**0.59**	**0.25**	**0.02**	**1.80**	**1.11, 2.91**

Note. n=940. Table includes results from multinomial logistic regression analyses that examined whether plasma biomarkers predict latent class group membership of self-reported functioning/cognitive performance profiles. Bonferroni correction was used for pairwise comparisons. Reference group LS/LC. *Abbreviations*: GFAP: glial fibrillary acidic protein, NfL: Neurofilament light chain, T-tau: total tau, UCH-L1: Ubiquitin C-terminal hydrolase L1, HS: high self-reported functioning, MS: moderate self-reported functioning, LS: low self-reported functioning; HC: high cognitive performance, LC: low cognitive performance, OR: odds ratio, LL: lower limit; UL: upper limit.

© 2026 Dark HE et al. *JAMA Network Open*.

eFigure 1. Study Timeline and Exclusion Flowchart

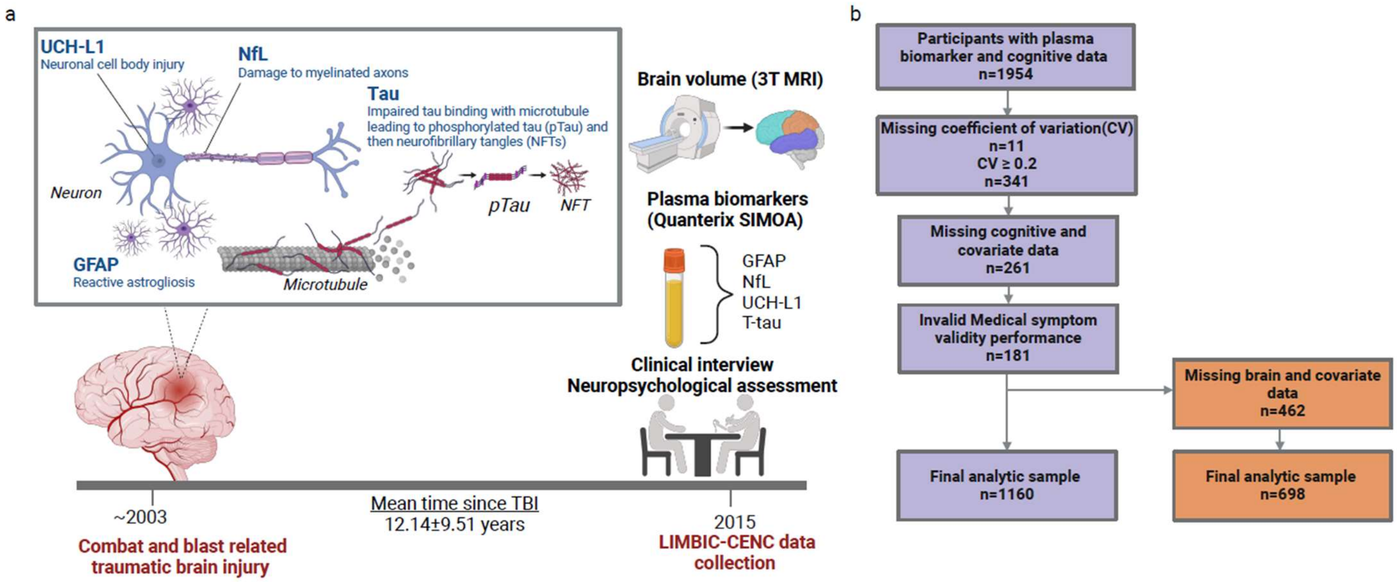

a) Enrollment data collection for LIMBIC-CENC began in 2015. Participants who experienced an mTBI in the present LIMBIC-CENC sample report experiencing an mTBI an average of approximately 12 years prior to data collection. Summary graphic depicting plasma biomarkers used in the present study and their cellular origin. During their LIMBIC-CENC enrollment visit, participants completed one T1 MRI scan, provided a blood sample from which biomarkers were derived, and completed a clinical interview and neuropsychological assessment. b) study exclusion flowchart. *Abbreviations*: GFAP: glial fibrillary acidic protein, NfL: Neurofilament light chain, T-tau: total tau, UCH-L1: Ubiquitin C-terminal hydrolase L1; CV: coefficient of variation. Created in BioRender. Dark, H. (2025) https://BioRender.com/ x1v4tcc.

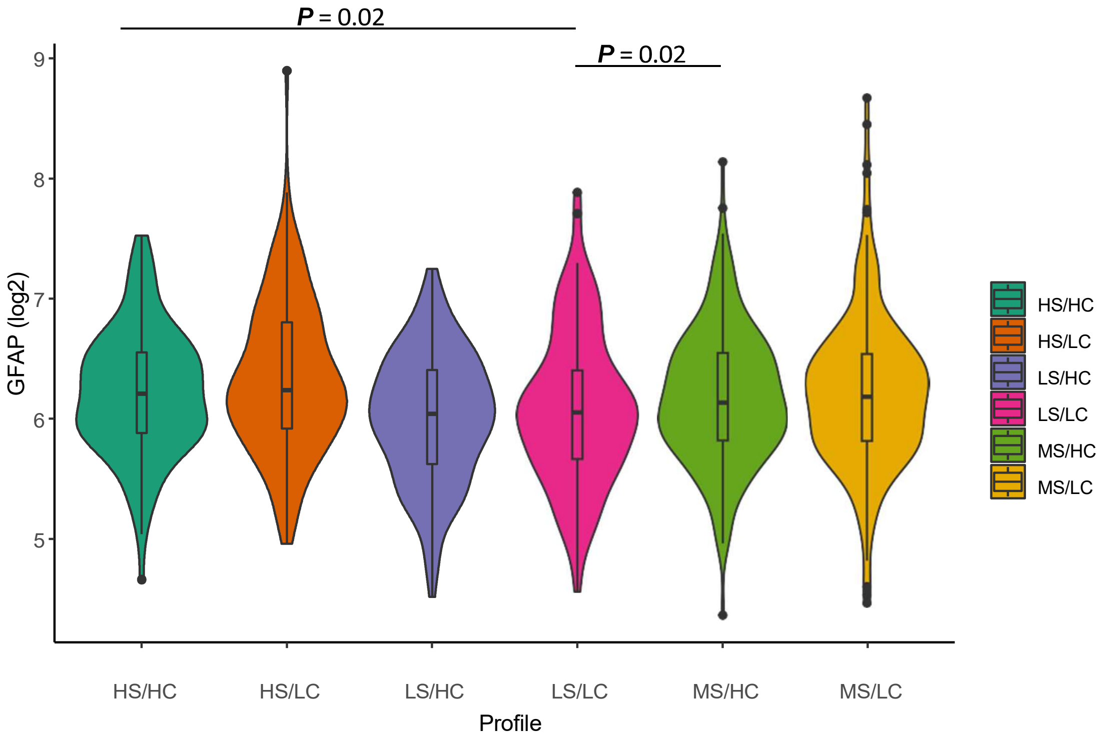

eFigure 2. GFAP (log2) by Self-Reported Functioning/Cognitive Performance Profile

Distribution of glial fibrillary acidic protein (GFAP; log2 transformed) by self-reported functioning/cognitive performance profile based on multinomial logistic regression in eTable 8. HS/HC: n=131, HS/LC: n=147, LS/HC: n=133, LS/LC: n=94, MS/HC: n=204, MS/LC: n=195. *Abbreviations*: GFAP: glial fibrillary acidic protein, HS: high self-reported functioning, MS: moderate self-reported functioning, LS: low self-reported functioning; HC: high cognitive performance, LC: low cognitive performance.

© 2026 Dark HE et al. *JAMA Network Open*.

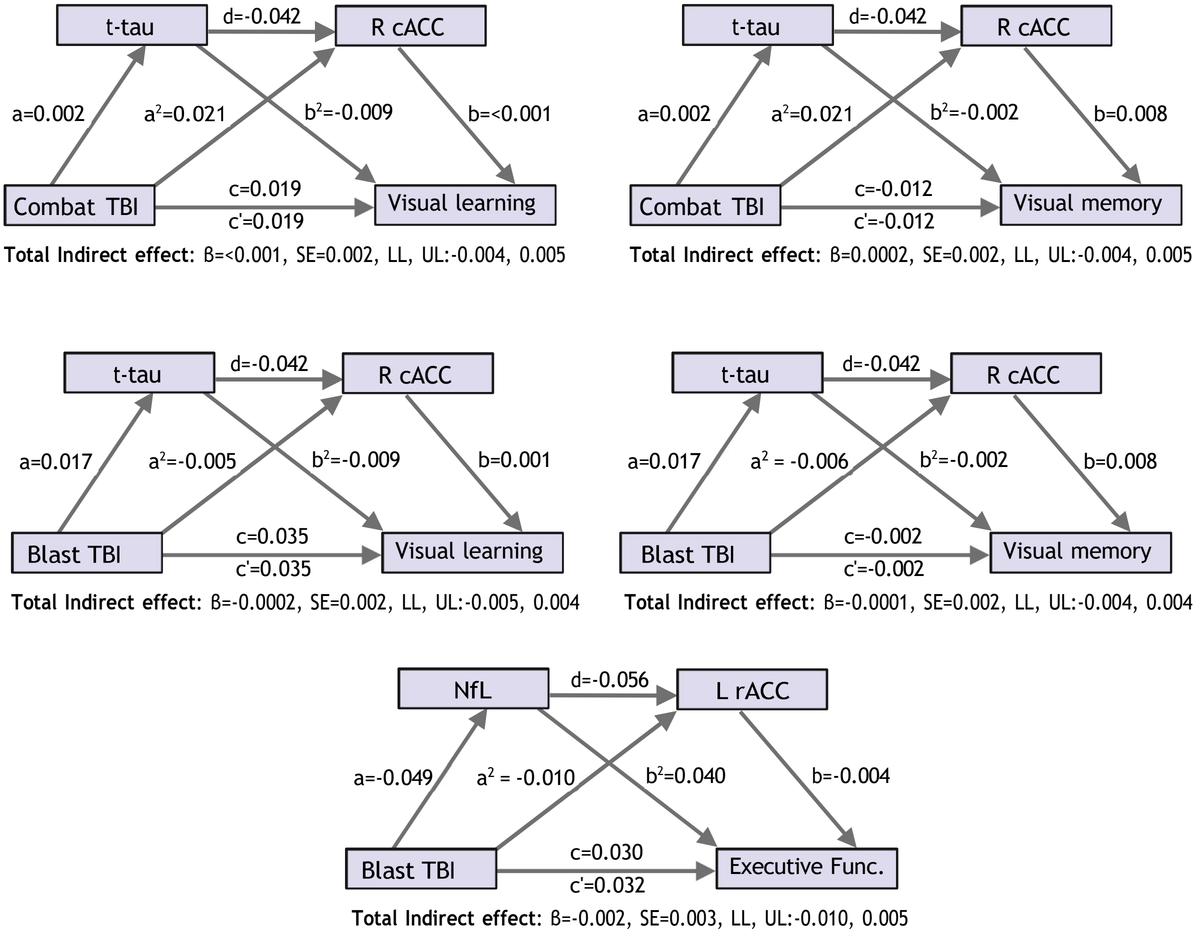

eFigure 3. Serial Mediation Analyses
a, a^2 = paths a; b, b^2 = paths b; c = total effect; c’ = direct effect; d = path d. Confidence intervals are based on 10,000 bootstrapped samples; n=698. Covariates for all analyses include age, sex, race, education, time since mTBI, and total intracranial volume. Coefficients are standardized. Abbreviations: t-tau: total tau; R cACC: right caudal anterior cingulate cortex; L rACC: left rostral anterior cingulate cortex; NfL: neurofilament light chain. Created in BioRender. Dark, H. (2025) https://BioRender.com/ x1v4tcc.

eReferences.

1. Vogt D, Smith BN, King LA, King DW, Knight J, Vasterling JJ. Deployment risk and resilience inventory-2 (DRRI-2): an updated tool for assessing psychosocial risk and resilience factors among service members and veterans. J Trauma Stress. 2013;26(6):710-7.
2. Walker WC, Carne W, Franke LM, Nolen T, Dikmen SD, Cifu DX, et al. The Chronic Effects of Neurotrauma Consortium (CENC) multi-centre observational study: Description of study and characteristics of early participants. Brain Inj. 2016;30(12):1469-80.
3. Green P. Medical Symptom Validity Test (MSVT) for microsoft windows: User's manual: Paul Green Pub.; 2004.
4. U.S. Department of Veterans Affairs, Department of Defense. *Clinical Practice Guideline for the Management of Concussion-Mild Traumatic Brain Injury*. Version 2.0. 2016.
5. Wechsler D. Wechsler Adult Intelligence Scale–Fourth Edition (WAIS-IV): NCS Pearson; 2008.
6. Reitan RM. Validity of the Trail Making Test as an Indicator of Organic Brain Damage. Perceptual and Motor Skills. 1958;8(3):271-6.
7. Delis DC, Kaplan E, Kramer JH. Delis-Kaplan executive function system. Assessment. 2001.
8. Benedict RH, Schretlen D, Groninger L, Dobraski M, Shpritz B. Revision of the Brief Visuospatial Memory Test: Studies of normal performance, reliability, and validity. Psychological assessment. 1996;8(2):145.
9. Delis DC, Kramer JH, Kaplan E, Ober BA. California verbal learning test. Assessment. 2000.
10. de Souza NL, Lindsey HM, Dorman K, Dennis EL, Kennedy E, Menefee DS, et al. Neuropsychological Profiles of Deployment-Related Mild Traumatic Brain Injury: A LIMBIC-CENC Study. Neurology. 2024;102(12):e209417.
11. Hayes AF. Introduction to mediation, moderation, and conditional process analysis : a regression-based approach. Third edition. ed: New York : The Guilford Press; 2022.

© 2026 Dark HE et al. *JAMA Network Open*.
