# Supplementary material for: Plasma Biomarkers, Brain Volume, and Cognitive Performance in Service Members and Veterans With mTBI: A LIMBIC-CENC Study
Source: JAMA Netw Open. 2026 Feb 25;9(2):e2559596. doi: 10.1001/jamanetworkopen.2025.59596 (PMC12936882; doi:10.1001/jamanetworkopen.2025.59596)
Supplement: Supplement 2. — Data Sharing Statement [file jamanetwopen-e2559596-s002.pdf]

Data Sharing Statement

Dark. Plasma Biomarkers, Brain Volume, and Cognitive Performance in Service Members and Veterans With mTBI:. *JAMA Netw Open*. Published February 20, 2026.
doi:10.1001/jamanetworkopen.2025.59596

Data
Data available: Yes
Data types: Deidentified participant data
How to access data: Access to the data for this study will be possible on request and approval by the LIMBIC-CENC data board (Virginia Commonwealth University, Richmond, Virginia).
When available: With publication

Supporting Documents
Document types: Other (please specify)
Additional Information: None
How to access documents: None
When available: With publication

Additional Information
Who can access the data: N/A
Types of analyses: N/A
Mechanisms of data availability: N/A
